# Complete Genome Sequence of *Entomomonas* sp. Strain E2T0, Isolated from the Darkling Beetle Zophobas morio Larvae

**DOI:** 10.1128/mra.01176-22

**Published:** 2022-12-21

**Authors:** Aline I. Moser, Yasmine Eddoubaji, Edgar I. Campos-Madueno, Andrea Endimiani

**Affiliations:** a Institute for Infectious Diseases (IFIK), University of Bern, Bern, Switzerland; b Graduate School of Cellular and Biomedical Sciences, University of Bern, Bern, Switzerland; University of Maryland School of Medicine

## Abstract

Here, we present the complete genome sequence of *Entomomonas* sp. E2T0, a strain isolated from larvae of the darkling beetle Zophobas morio. The isolate was fully resistant to aztreonam and possessed a novel class D β-lactamase gene. The 3,325,929-bp genome consists of a chromosome and a 9,996-bp plasmid.

## ANNOUNCEMENT

Members of the genus *Entomomonas* are associated with the digestive tract of insects (1). To date, only two *Entomomonas* spp. have been sequenced (1, 2). The antibiotic resistance profiles of these two strains have not been determined, but both strains possessed uncharacterized β-lactamase genes. Here, we analyzed the complete genome sequence of *Entomomonas* sp. strain E2T0, isolated from larvae of the darkling beetle Zophobas morio.

Larvae were obtained in October 2021 from a pet shop in Bern, Switzerland. *Entomomonas* sp. strain E2T0 was isolated from homogenized larva tissue plated onto selective ChromID ESBL plates (bioMérieux) after incubation at 36°C for 72 hr. Antimicrobial susceptibility tests performed using the GNX2F and ESB1F Sensititre panels (Thermo Scientific) indicated that the strain was resistant to cefoxitin and aztreonam (Table 1). Strain E2T0 was reactivated from a 20% glycerol stock at −80°C by plating it onto Columbia agar with 5% sheep blood (citrated sheep blood agar [CSBA]; Oxoid) at 36°C for 48 hrs. DNA was isolated using the PureLink microbiome DNA purification kit (Invitrogen). Whole-genome sequencing (WGS) was achieved by combining short- and long-read sequencing. Short reads were obtained with the NovaSeq 6000 platform using the NEBNext Ultra II DNA library prep kit for Illumina (2 × 150-bp paired-end reads), and long reads were obtained with the MinION device using the SQK-RBK004 library and FLO-MIN 106D R9 flow cells (Oxford Nanopore Technologies [ONT]) and subsequent basecalling with Guppy v3.4.5 (ONT; high-accuracy model). Sequencing adapters were removed with Trimmomatic v0.36 and Porechop v0.2.4 for the Illumina and Nanopore reads, respectively (3, 4). A *de novo* assembly was generated using Unicycler v0.4.8 and annotated using the NCBI Prokaryotic Genome Annotation Pipeline (5). Circularity and completeness of the assembled genome were confirmed by the Unicycler pipeline and, in addition, by mapping Illumina reads and an independent Illumina short-read assembly (SPAdes v3.14) to the complete hybrid genome (Bowtie 2 v2.3.4.1) (6, 7). The completed circular contigs were not rotated by Unicycler. The final genome was analyzed with the Resistance Gene Identifier (RGI) and compared with similar genomes using BLASTn and the average nucleotide identity (ANI) calculator OrthoANIu (www.ezbiocloud.net/tools/orthoaniu/) (8, 9). All software was run with default parameters.

**TABLE 1:** Antimicrobial susceptibility tests for the *Entomomonas* sp. E2T0 strain

Antibiotics	MIC value (mg/L)^*a*^
Piperacillin-tazobactam	≤8/4 (S)
Ticarcillin-clavulanate	≤16/2 (S)
Cefoxitin	64 (R)
Ceftazidime	0.5 (S)
Ceftazidime-clavulanate	0.25 (NA)
Ceftriaxone	≤1 (S)
Cefotaxime	0.5 (S)
Cefotaxime-clavulanate	0.25 (NA)
Cefepime	≤1 (S)
Aztreonam	>16 (R)
Imipenem	≤1 (S)
Meropenem	≤1 (S)
Doripenem	≤0.12 (S)
Ertapenem	≤0.25 (S)
Gentamicin	≤1 (S)
Tobramycin	≤1 (S)
Amikacin	≤4 (S)
Ciprofloxacin	≤0.25 (S)
Levofloxacin	≤1 (S)
Doxycycline	≤2 (NA)
Minocycline	≤2 (NA)
Tigecycline	≤0.25 (S)
Trimethoprim-sulfamethoxazole	≤0.5/9.5 (S)
Colistin	≤0.25 (S)
Polymyxin B	≤0.25 (NA)

a MICs were obtained with microdilution Sensititre panel GNX2F and ESB1F. MIC results were interpreted according to the 2022 European Committee on Antimicrobial Susceptibility Testing (EUCAST) criteria for *Enterobacterales* (https://www.eucast.org/; version 12.0). For cefoxitin, the Clinical and Laboratory Standards Institute (CLSI) 2022 criteria were used (document M100-S32). Antibiotics for which strain E2T0 was resistant are highlighted in gray. R, resistant; S, susceptible; NA, not available.

A total of 32,745 Nanopore (*N*_50_, 17,387 bp) and 10,786,558 Illumina reads were obtained after sequencing. The complete genome consisted of a 3,315,933-bp chromosome and a 9,996-bp plasmid with an average coverage of 474× and a GC content of 35.9%. The annotated genome contained 3,054 protein-coding sequences (CDSs), 12 rRNAs, and 47 tRNAs. The 16S rRNA gene sequence of strain E2T0 was most similar to the one from Entomomonas asaccharolytica F2A^T^ (GenBank accession no. CP067393) (98.2% identity) and Entomomonas moraniae QZS01^T^ (GenBank accession no. CP029822) (97.3% identity). The ANI between E2T0 and these two strains was 88.40% and 75.34% for F2A^T^ and QZS01^T^, respectively, implying that our strain belongs to the genus *Entomomonas*. E2T0 possessed a class D β-lactamase (CDS MTZ49_05555 in GenBank accession no. CP094972) which showed the best amino acid homology with the putative class D β-lactamase of *E. asaccharolytica* F2A (82.76% identity) (GenPept accession no. WP_201091896.1). Characterization of this novel class D β-lactamase could provide new insights into the evolution and natural reservoirs of oxacillinase enzymes.

### Data availability.

The complete genome sequence of *Entomomonas* sp. E2T0 has been deposited in GenBank (CP094972 and CP094973) under BioProject PRJNA821407. The version described in this paper is the first version. The raw reads are available in the Sequence Read Archive (SRA) under SRR22077681 and SRR22077682 for Illumina and Nanopore, respectively.
